# FXR induces SOCS3 and suppresses hepatocellular carcinoma

**DOI:** 10.18632/oncotarget.5314

**Published:** 2015-09-21

**Authors:** Fei Guo, Zhizhen Xu, Yan Zhang, Peng Jiang, Gang Huang, Shan Chen, Xilin Lyu, Ping Zheng, Xin Zhao, Yijun Zeng, Shuguang Wang, Fengtian He

**Affiliations:** ^1^ Department of Hepatobiliary Surgery Institute, Southwest Hospital, Third Military Medical University, Chongqing 400038, China; ^2^ Department of Biochemistry and Molecular Biology, College of Basic Medical Sciences, Third Military Medical University, Chongqing 400038, China

**Keywords:** HCC, FXR, SOCS3, STAT3

## Abstract

Suppressor of cytokine signaling 3 (SOCS3) is regarded as a vital repressor in the liver carcinogenesis mainly by inhibiting signal transducer and activator of transcription 3 (STAT3) activity. Farnesoid X Receptor (FXR), highly expressed in liver, has an important role in protecting against hepatocellular carcinoma (HCC). However, it is unclear whether the tumor suppressive activity of FXR involves the regulation of SOCS3. In the present study, we found that activation of FXR by its specific agonist GW4064 in HCC cells inhibited cell growth, induced cell cycle arrest at G1 phase, elevated p21 expression and repressed STAT3 activity. The above anti-tumor effects of FXR were dramatically alleviated by knockdown of SOCS3 with siRNA. Reporter assay revealed that FXR activation enhanced the transcriptional activity of *SOCS3* promoter. Electrophoretic mobility shift assay (EMSA) and chromatin immunoprecipitation (ChIP) assay displayed that FXR directly bound to IR9 DNA motif within *SOCS3* promoter region. The *in vivo* study in nude mice showed that treatment with FXR ligand GW4064 could decelerate the growth of HCC xenografts, up-regulate SOCS3 and p21 expression and inhibit STAT3 phosphorylation in the xenografts. These results suggest that induction of SOCS3 may be a novel mechanism by which FXR exerts its anti-HCC effects, and the FXR-SOCS3 signaling may serve as a new potential target for the prevention/treatment of HCC.

## INTRODUCTION

The signal transducer and activator of transcription 3 (STAT3) has been implicated in intracellular signaling transduction by different cytokines and growth factors. In normal cells, STAT3 activation is tightly controlled to prevent dysregulated gene transcription, whereas hyperactivation of STAT3 plays an important role in tumor cell proliferation, survival, invasion and immunosuppression, in diverse types of human cancers including hepatocellular carcinoma (HCC) [1, 2]. More and more evidences reveal that blocking STAT3 activation is beneficial for cancer therapy [3–6]. Suppressor of cytokine signaling 3 (SOCS3), which is a physiologic negative regulator of STAT3, has received increasing attention. Some pharmacologically safe and effective compounds including MicroRNA let7, nuclear receptor peroxisome proliferators-activated receptor gamma (PPAR γ) ligand, and angiostatic chemokine platelet factor-4 have already been reported to block STAT3 activation through enhancing expression of SOCS3 [7–9].

SOCS3 can be induced strongly by a variety of cytokines and other stimulators, then act to negatively regulate Janus tyrosine kinase 2 (JAK2) -STAT3 activity by inactivating JAK2 or blocking recruitment sites for STAT3 and also may target signaling complexes for ubiquitination and degradation [10, 11]. Loss of SOCS3 in HCC is associated with STAT3 over phosphorylation and poor prognosis [12–14]. Accumulating studies support that inhibition of SOCS3 expression promoted STAT3 activation, enhanced hepatic fibrosis, increased proliferation and tumor aggressiveness [12, 15, 16]. Moreover, the importance of STAT3 to hepatocarcinogenesis was illustrated by the fact that hepatocyte-specific deletion of SOCS3 in a mouse HCC model results in larger and more numerous tumors [17]. So restoration of SOCS3 should be a potential therapeutic strategy for HCC prevention and treatment. To further understand the contribution of SOCS3 to HCC progression, it is essential to better define the detailed regulatory mechanism of SOCS3 expression.

Farnesoid X receptor (FXR; NR1H4), a member of the nuclear receptor superfamily, is highly expressed in the gut-liver axist. FXR can be activated by a wide variety of compounds such as chenodeoxycholic acid (CDCA) and specific synthetic agonist GW4064 [18]. The ligand-activated FXR binds to its responsive elements as a classical heterodimer with retinoid X receptor alpha (RXR α) or as a monomer to coordinating the expression of target genes [19, 20]. In recent years, the understanding of the role of FXR in the liver has developed from that as a metabolic regulator to the novel function as a cell protector required for participating in carcinogenesis including promoting liver regeneration, suppressing hepatic inflammation, and regulating cell growth and differentiation, and thus may be a promising target for the prevention/treatment of liver cancer [21]. However, it is not well known whether the anti-tumor effect of FXR involves the regulation of SOCS3 or STAT3.

In our previous study, we have demonstrated that FXR activation led to an increased expression of SOCS3 in hepatocytes and mice liver, then to protect against the development of LPS-induced liver inflammation [22]. Furthermore, in the present study we continued to investigate the ability of FXR to influence HCC cell growth through the up-regulation of SOCS3 and the inhibition of STAT3 activation. This study will advance our understanding of the molecular mechanism of liver carcinogenesis targeting FXR and SOCS3.

## RESULTS

### FXR agonist GW4064 inhibits HCC cells growth and induces cell cycle arrested

**Figure 1:**
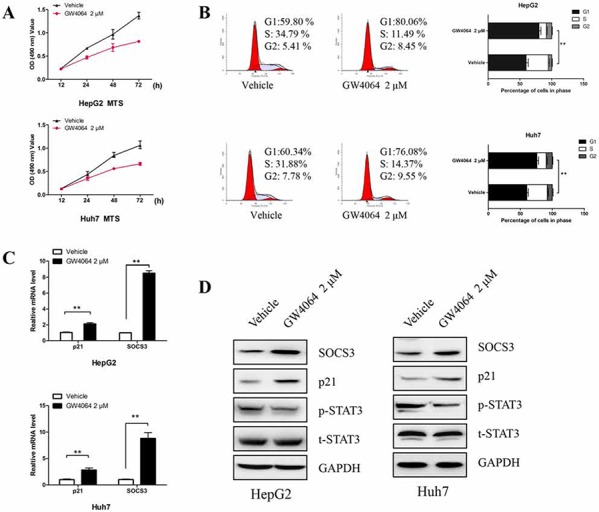
Activation of FXR in HCC cells inhibits cell growth, induces cell cycle arrest, up-regulates SOCS3 and p21, and represses STAT3 activation **A.** HepG2 cells and Huh7 cells were treated with vehicle DMSO (0.1%) or GW4064 (2 μM) for different times, and then the cell proliferation was determined with MTS assay. Data are means ± SEM from three independent experiments in triplicate. **B–D.** HepG2 cells and Huh7 cells were treated with GW4064 (2 μM) or vehicle DMSO for 24 h. Then the cell cycle was analysed using PI staining and flow cytometry. The percentage of cells in each cell cycle phases was quantified (B). The mRNA levels of SOCS3 and p21 were examined by RT-qPCR, taking GAPDH as a control. Data are means of three separated experiments ± SEM, **P < 0.01 (C). The protein levels of SOCS3, p21, total STAT3 (t-SATA3) and phosphoraylated STAT3 (p-STAT3) were assayed by Western blot, taking GAPDH as a loading control (D).

The elucidation of the mechanism how FXR controls the proliferation of HCC cells is useful to establish the therapy for liver cancer. MTS assay revealed that FXR specific agonist GW4064 drastically decreased the rate of cell proliferation of HepG2 cells and Huh7 cells when compared with the corresponding control on the 48 or 72 h (Fig. 1A). Meanwhile as shown in Fig. 1B, the GW4064-mediated growth inhibition of HCC cells was associated with suppressing entry into the S phase. We also examined the effect of GW4064 on the alterations of p21 expression which controls cell proliferatrion and cell cycle progression. There was an obvious up-regulation in p21 at both transcriptional and translational levels. Besides, upon FXR activation, STAT3 activation, as assessed by the amount of Tyr705-phosphorylated protein, was inhibited, whereas total STAT3 protein remained unchanged (Fig. 1C and 1D). And we also observed that these events accompanied by an increased expression of SOCS3.

### Up-regulation of SOCS3 is involved in the FXR-mediated cell growth repression in HCC cells

**Figure 2:**
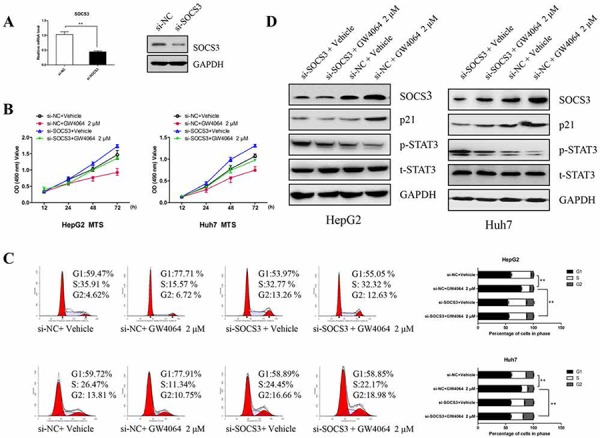
GW4064 influences cell proliferation in HCC cells partly through SOCS3 **A.** Huh7 cells were transiently transfected with the siRNA targeting SOCS3 for 48 h, the expression of SOCS3 was examined by RT-qPCR and Western blot. **B.** After silencing SOCS3 expression for 48 h, HepG2 cells and Huh7 cells were treated with GW4064 (2 μM) or vehicle DMSO for different times. Then the cell proliferation was detected by MTS assay. Data are means ± SEM from three experiments in triplicate. **C.** After interfering SOCS3 expression for 48 h, the cells were treated by GW4064 (2 μM) or vehicle DMSO for another 24 h. Then the percentage of cells in each cell cycle phases was determined. Data are means of three separated experiments ± SEM, **P < 0.01. **D.** Western blot were assayed for the expression of SOCS3 and p21 and the phosphorylation of STAT3.

A recently study has demonstrated that SOCS3 regulated p21 gene expression and induced cell cycle arrest primarily through its negative regulation of STAT3 signaling [23]. To investigate whether SOCS3 is involved in the anti-proliferative effect of FXR, we decreased SOCS3 expression through siRNA approach (Fig. 2A). As expected, Fig. 2B and 2C showed that the effect of GW4064-induced cell proliferation inhibition and cell cycle arrest at G1 phase were markedly relieved by disruption of the SOCS3 gene. Knockdown of SOCS3 also attenuated phosphorylation of STAT3 and the expression levels of p21 (Fig. 2D). These observations convincingly suggest that SOCS3 plays an important role in the FXR-mediated anti-HCC effects. Namely, FXR exerts the inhibitory ability on HCC, at least partially, through induction of SOCS3.

### IR9 is a likely FXRE within the human *SOCS3* promoter region

**Figure 3:**
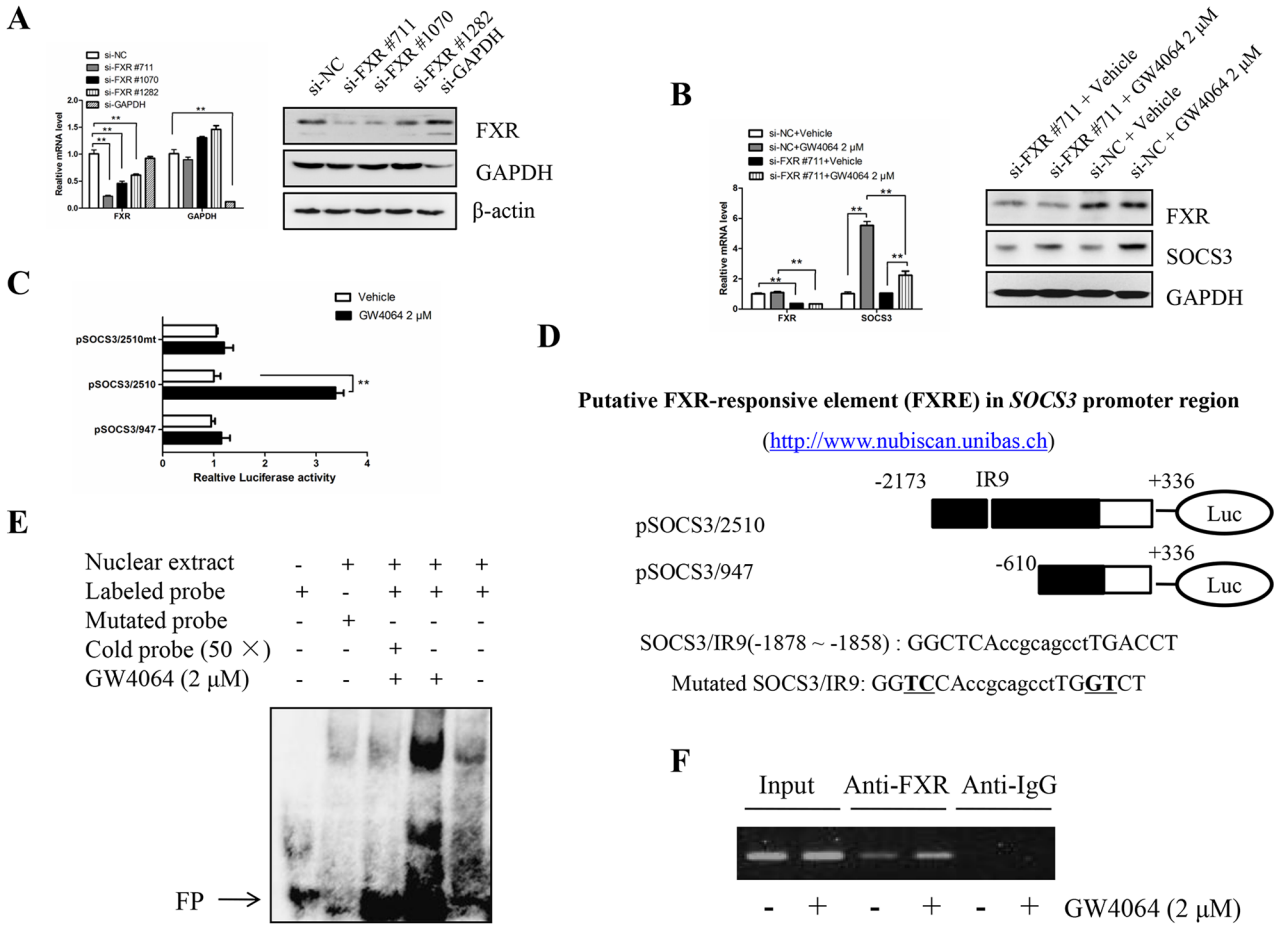
FXR binds to FXRE in human *SOCS3* promoter region **A.** Huh7 cells were transiently transfected with the siRNAs for FXR (targeting 711, 1070, 1282 sites respectively), the expression of FXR was examined by RT-qPCR and Western blot 48 h after transfection, and the one (numbered as si-FXR#711) with the best silence effect was selected to be used in the subsequent experiments. The siRNA targeting GAPDH is a positive control. **B.** After interfering FXR expression (si-FXR#711) for 48 h, Huh7 cells were treated with GW4064 (2 μM) or vehicle DMSO for another 24 h, RT-qPCR and Western blot were assayed for FXR and SOCS3 expression. Data are means of three separated experiments ± SEM, **P < 0.01. **C.** HepG2 cells were transiently transfected with the luciferase reporter plasmid pSOCS3/2510, pSOCS3/947 or pSOCS3/2510mt, followed by treatment with GW4064 (2 μM) for 24 h. Then the luciferase assay was performed. Results represent three independent experiments performed in triplicate. **P < 0.01. **D.** Prediction of a putative FXRE in human *SOCS3* promoter region via an in silico analysis with a Web-based algorithm (NUBIScan). **E.** EMSA for analyzing the binding between the FXRE/IR9 probe and the nuclear proteins derived from HepG2 cells treated with vehicle DMSO or GW4064 (2 μM) for 24 h. FP: free probe. **F.** ChIP assay with chromatin isolated from HepG2 cells treated with vehicle DMSO or GW4064 (2 μM) for 24 h. Antibody directed against FXR was used for immune precipitation, taking IgG as a negative control. Final DNA extractions were amplified by PCR with the primer pairs covered the IR9 sequence in *SOCS3* promoter region. Total extract (input) was used as positive PCR control.

The effect of GW4064-mediated SOCS3 induction was decreased in the presence of the specific FXR siRNA, further supporting the direct involvement of this nuclear receptor (Fig. 3A and 3B). As a classical transcriptional factor, FXR usually regulates the transcription of target genes via directly binding to FXR-responsive element (FXRE). As shown in Fig. 3C, treatment with GW4064 had an apparent stimulatory effect on the *SOCS3* promoter (pSOCS3/2510) transcriptional activity. However, the transcriptional activity was dramatically diminished when the sequence (−2173 to −610) in the *SOCS3* promoter region was deleted (pSOCS3/947) and resulted in failure in response to GW4064, which suggested that this DNA fragment in the region might harbor a key positive regulatory element which is sufficient for SOCS3 transcriptional activation. Sequence analysis of the *SOCS3* promoter region with a Web-based algorithm (NUBIScan) predicted a potential FXRE/IR9 (an inverted repeat spaced by nine nucleotides, −1878 to −1858) in the human *SOCS3* promoter region, and its sequence and location were shown in Fig. 3D.

Moreover, the mutation of FXRE/IR9 in *SOCS3* promoter region abolished the GW4064-induced luciferase activity, indicating that FXRE/IR9 is very vital for FXR-enhanced transcriptional activation of *SOCS3* gene promoter (Fig. 3C, pSOCS3/2510mt). To determine whether FXR directly binds to this element, EMSA and ChIP were performed. The sequences of the probes used in EMSA were showed in Fig. 3D. Interaction of SOCS3/IR9 probe with the nuclear extract of HepG2 cells yielded a DNA/protein band of expected mobility. The binding was greatly increased when the nuclear extract derived from GW4064- treated (Fig. 3E). Furthermore, this binding was sequence specific because (1) the binding was significantly weakened by a 50-fold excess of the unlabeled IR9 probe (cold probe); and (2) there was no obvious interaction between the nuclear extract and the mutated IR9 probe. Subsequently, ChIP assays showed that FXR could directly bind to the FXRE/IR9 in *SOCS3* promoter region in HepG2 cells, and the binding was markedly enhanced after treatment with GW4064 (Fig. 3F). The above results suggest that FXR induces SOCS3 expression via directly and specifically binding to the FXRE/IR9.

### FXR agonist suppresses HCC xenograft and represses STAT3 activation *in vivo*

**Figure 4:**
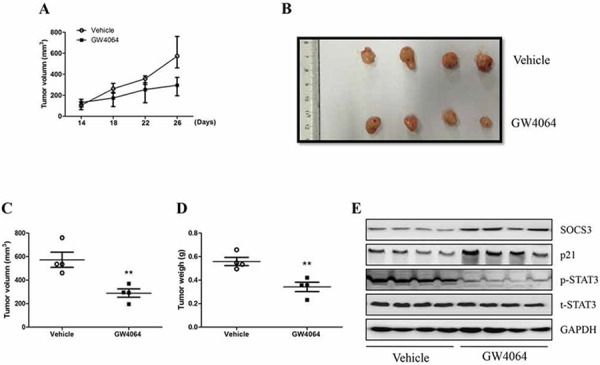
FXR ligand GW4064 represses the growth of HCC xengrafts and inhibits STAT3 activation *in vivo* HepG2 cells (1.0 × 10^6^/per mouse) were injected subcutaneously into the flank of nude mice. When palpable tumor were formed, GW4064 (25 mg/kg/day) or vehicle DMSO alone were administrated by intraperitoneally injected once every two day for 2 weeks. **A.** Tumor gowth was monitored over time. **B–D.** Tumors were photographed and harvested for analysis of the differences of tumor size and mass. Data are means ± SEM, *n* = 4, **P < 0.01. **E.** Tumor samples were subjected to quantitative western blot analysis for SOCS3 and p21expression and STAT3 activation.

The above studies clearly clarified that FXR induced SOCS3 expression and this induction played an important role in FXR-mediated growth suppression of HCC cells *in vitro*. Then we examined the influence of FXR agonist on tumor growth and SOCS3 expression in HCC tumor xenograft model. We injected HepG2 cells into the intrascapular region of nude mice and followed by administration of GW4064. In consistent with the above *in vitro* findings, we observed that GW4064 induced a regression in tumor growth during this period (Fig. 4A). As shown in Fig. 4B–4D, tumor size and mass were noticeably decreased in mice treated with GW4064 compared with the vehicle-treated group, at the time of killing day. Remarkably, this was accompanied by the up-regulation of SOCS3 and p21 expression in the xenograft tumors, the phosphorylation of STAT3 was lessened as well (Fig. 4E). These data suggest that FXR agonist can exert anti-HCC effects via up-regulating SOCS3 expression *in vivo*.

### FXR and SOCS3 expression levels are positively correlated in human HCC specimens

**Figure 5:**
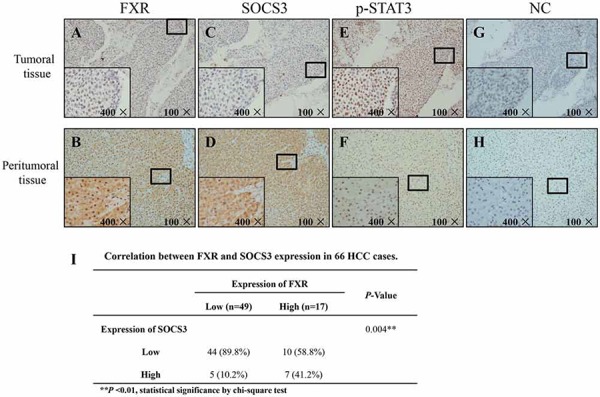
FXR and SOCS3 expression levels are positively correlated in human HCC specimens Expression profile of FXR and SOCS3 and activation of STAT3 on formalin-fixed paraffin-embedded clinical samples were investigated by Immunohistochemistry. **A–H.** Representative images are shown. Negative control (NC): the primary antibody was replaced by rabbit IgG (isotype control). **I.** Correlation between FXR and SOCS3 expression in 66 HCC cases. **P < 0.01, Statistical significance by chi-square test.

We examined the expression profile of FXR and SOCS3 and the activation of STAT3 in 66 HCC samples and the corresponding peritumoral tissues using IHC assay (Fig. 5). Statistical analysis with chi-square test showed that there was a positive correlation between FXR and SOCS3 expression (Fig. 5I, *P* < 0.01). Additionally, tumoral FXR and SOCS3 expression were significantly lower than that of the peritumoral tissue, whereas STAT3 was over-activated in HCC lesions, indicating that the dysexpression of FXR and SOCS3 might be involved in the development and/or progression of HCC.

## DISCUSSION

Several studies support a role for SOCS3 as a tumor suppressor in different types of cancers [24, 25]. Increasing evidences have displayed over-expression of SOCS3 exhibited preclinical anti-tumor activity against HCC, malignant pleural mesotheliom and inflammation-associated colon cancer [26–28]. Up-regulation of SOCS3 will no doubt aid to the development of novel therapeutic strategies. In this study, we illustrate that FXR activation represses STAT3 activation and increases p21 expression via induction of SOCS3, then influences the cell cycle to suppress HCC cells growth. In addition, activation of FXR reduces the *in vivo* tumor growth rate in a mouse xenograft model. Taken together, these results suggest that FXR may serve as a key transcriptional regulator of cell proliferation in HCC by stimulating SOCS3 expression, and FXR-SOCS3 pathway may be a novel target for the treatment/prevention of HCC.

In the previous study, we for the first time discovered that both SOCS3 mRNA and protein were highly induced in HepG2 cells and mice livers in response to FXR ligand(s) [22]. Reporter assay revealed that SOCS3 promoter activity was significantly increased with FXR agonist treatment. As a classical transcriptional factor, FXR usually regulates the transcription of target genes via directly binding to FXRE such as IR1 (inverted repeat separated by 1 bp) [20], DR8 (directed repeat separated by 8 bp) [29], and ER8 (everted repeat separated by 8 bp) [30]. In this study, as summarized in Fig. 3, IR9 is identified as a novel FXRE that is involved in up-regulation of SOCS3 in HCC cells, which means SOCS3 is a new direct target gene of FXR. Besides, previous studies have demonstrated that another nuclear receptor PPAR γ could promote SOCS3 expression [8, 31], and FXR could up-regulate PPAR γ [32, 33]. So FXR may also indirectly enhance the expression of SOCS3 via inducing PPAR γ.

Given the great physiological importance in metabolism homeostasis, as well as in the regulation of inflammation and fibrosis, FXR plays a suppressive role in the liver carcinogenesis. Numerous studies showed that mice displayed spontaneous development of liver tumors in the absence of FXR [22, 34–38], we have reported that the inhibition of FXR promoted the proliferation, migration, and invasion of HCC cells [39]. FXR can protect against HCC by several mechanisms, including antagonizing nuclear factor-κB (NF-κB) activation to less hepatic inflammatory response [40], inhibiting a small subunit of proteasome gankyrin to subsequent protection of tumor suppressor proteins (Rb, p53, HNF4α) from degradation [41] or interacting with Wnt/β-catenin activation and E-cadherin expression [42, 43]. FXR directly induces the expression of HCC suppressors such as SHP (small heterodimer partner) [35] and NDRG2 (N-myc downstream regulated gene 2) [44] as well. Although multiple factors participate in the execution of FXR activation, its downstream genes that it regulates in HCC progression remains largely unexplored. Here we reported that SOCS3, the feedback inhibitor of the STAT3 pathway, was a direct FXR target gene. Knockdown of SOCS3 by siRNA substantially attenuated the GW4064-mediated repression of STAT3 activation and HCC cell growth. Our data displayed FXR-SOCS3 signaling as a novel mechanism in FXR-mediated anti-HCC effects. Although further research is needed, these studies strongly indicate that FXR is a negative modulator of hepatic cell hyperproliferation, ergo therapeutic modulation of FXR and SOCS3 could be profitable in patient with liver carcinoma, namely, which provides a new view about hepatic cancer treatment when targeting this FXR-SOCS3 signaling.

## MATERIALS AND METHODS

### Cell lines and cell culture

HCC cell lines HepG2 and Huh7 obtained from American Type Culture Collection were cultured in Dulbeco's Modified Eagle's medium (DMEM) supplemented with 10% fetal bovine serum (FBS) and 1% Penicillin/Streptomycin (complete medium) in a humidified atmosphere of 5% CO_2_ at 37°C. The cells grown to 70%–80% confluence were cultured in DMEM supplemented with 0.5% FBS and without antibiotics (conditioned medium), then treated with 2 μM FXR agonist GW4064 (Sigma–Aldrich, St. Louis, MO, USA) or vehicle dimethylsulphoxide (DMSO, 0.1%).

### Western blot analysis

HCC cells or mice xgnograft tumors were lysed and the protein concentrations were normalizated by the BCA protein assay (Beyotime Inst Biotech, Beijing, China). Western blot analysis was performed as described previously [21]. Rabbit anti-FXR antibody (ab28676), rabbit anti-SOCS3 antibody (ab16030), rabbit anti-GAPDH antibody (ab181602) and rabbit IgG (isotype control, ab172730) were purchased from Abcam (San Francisco, CA, USA). The rabbit antibodies for total STAT3 (t-STAT3, #9132) and phosphorylated STAT3 (p-STAT3, Tyr705, #9145) were from Cell Signaling Technology (Beverly, MA, USA). Rabbit anti-P21 antibody (10355–1-AP) was bought from Proteintech (Chicago, IL, USA). The enhanced chemiluminescence detection reagents (Pierce, Rockford, IL, USA) were used to visualize the signals.

### RNA extraction and Real-time quantitative polymerase chain reaction (RT-qPCR) assay

Total RNA was isolated with TRIzol reagent and the first-strand cDNA was synthesized using reverse transcriptase (Invitrogen, Carlsbad, CA, USA). The evaluation of gene expression was performed by real-time quantitative Polymerase Chain Reaction analysis. The mRNA levels of these genes were normalized to GAPDH mRNA levels. The primer sequences used for RT-qPCR are listed in Table 1.

**Table 1:** The primer sets for RT-qPCR

Gene	Product size (bp)	Forward primer	Reverse primer
SOCS3	136	5′-ATCCTGGTGACATGCTCCTC-3′	5′-GGCACCAGGTAGACTTTGGA-3′
p21	98	5′-CTGGAGACTCTCAGGGTGAA-3′	5′-GGATTAGGGCTTCCTCTTGGA-3′
GAPDH	159	5′-CAATGACCCCTTCATTGACC-3′	5′-GACAAGCTTCCCGTTCTCAG-3′

### Cell proliferation MTS assay

HepG2 cells and Huh7 cells were seeded in 96-well plates and the following day cells were treated with GW4064 (2 μM) or vehicle (0.1% DMSO). After 12, 24, 48, or 72 h, cell proliferation assay was performed using a MTS assay (Promega, Madison, WI, USA) according to the manufacturer's instructions. Briefly, 2 × 10^3^ cells per well were seeded in 96-well plates and cultured for overnight. Then, the cells were treated with GW4064 or DMSO. After 12, 24, 48, or 72 h, MTS reagent (20 μL) was added to the cells in each well followed by incubation for 2 h, and the absorbance was determined at 490 nm using a microplate reader.

### Cell cycle assay

After treated with GW4064 (2 μM) for 24 h, the harvested cells were fixed with 70% ethanol in PBS at −20°C overnight then stained with propidium iodide (PI) using Cell Cycle Phase Determination Kit (Cayman, Ann Arbor, MI, USA), and the samples were then analyzed for cell cycle phase distribution using a FACScan flow cytometer. The data were analyzed by using the Cell Quest computer program (BD).

### *SOCS3* gene silencing by small interfering RNA (siRNA)

The siRNA sequences used for targeting human *SOCS3* (sense 5′-CCAAGAACCUGCGCA UCCAdTdT-3′; antisense, 5′-UGGAUGCGCAGGUUCUUGGdTdT-3′) were synthesized by Genepharma (Shanghai, China). A non-targeting siRNA pool was used as a negative control (NC). The experiment details were performed as previous description [22].

### Plasmid vector construction and luciferase assay

Human *SOCS3* promoter region containing fragments (−2173 to +336 and −610 to +336) were amplified by PCR using genomic DNA of Huh7 cell as template. The primer sequences used for plasmid construction are listed in Table 2. The fragments were digested with *Kpn I* and *Nhe I* then cloned into pGL3-basic vector and the resulting plasmid were named as pSOCS3/2510 and pSOCS3/947. Site-directed mutation in pSOCS3/2510 at the IR9/FXRE (−1878 to −1858 from GGCTCAccgcagcctTGACCT to GGTCCAccgcagcctTGGTCT, the mutated bases were underlined) was constructed by employing TaKaRa mutant BEST Kit (TaKaRa, Dalian, China), and the resulting plasmid was named as pSOCS3/2510mt. Cell extracts were prepared after transfection, the luciferase and β-galactosidase (β-gal) activity assays were performed as described [45].

**Table 2:** The primer sets for plasmids construction

Plasmid	Forward primer	Reverse primer
pSOCS3/2510	5′- ggGGTACCTCTGACTCCCTGGTTCAAGC-3′	5′- cgGCTAGCCTTCCTACCTGGTCCCGAAT-3′
pSOCS3/947	5′- ggGGTACCACTGTCGCACGTCTCCAAC-3′	5′- cgGCTAGCCTTCCTACCTGGTCCCGAAT-3′

### Electrophoretic mobility shift assay (EMSA)

The EMSA was performed using the LightShift chemiluminescent EMSA kit (Pierce, Rockford, IL, USA), details were performed as previous description [45]. The DNA probe (SOCS3/IR9, 5′-GATCAT GGCTCAccgcagcctTGACCTCCCT-3′), containing a putative FXR response element (the underlined nucleotides), was derived from human *SOCS3* promoter region and was end-labeled with biotin. The mutated probe (5′-GATCATGGTCCAccgcagcct TGGTCTCCCT-3′, the mutant bases were underlined) was also included. For competition experiments, the corresponding unlabeled oligonucleotide (cold probe) was used at 50 × excess concentrations over the labeled probe in the binding reaction.

### Chromatin immunoprecipitation assay (ChIP)

ChIP assay was performed using a ChIP assay kit (Upstate Biotechnology, Lake Placid, NY, USA) as described previously [45]. Final DNA extractions were PCR amplified using the primer pairs that covered the putative FXRE/IR9 sequence in the *SOCS3* promoter region (forward primer, 5′ -TCTCACTCTGTTGCCCAGAC-3′; reverse primer, 5′-GTGGCCTGTGCCTGTAGTC-3′).

### Xenotransplantation of HCC cells in nude mice

HepG2 cells (1.0 × 10^6^/per mouse) in 100 μL phosphate buffered saline (PBS) were injected subcutaneously into the flank of 6-week-old male nude mice (4 mice/group). When palpable tumor were formed (about 2 weeks), GW4064 (25 mg/kg/day) or Vehicle DMSO treatment were started by intraperitoneally injected once every two day for 2 weeks. Tumor growth was monitored by caliper measurements along two orthogonal axes as described [46]. Tumor volume was calculated as V (mm^3^) = (length × width^2^)/2. Tumors were carefully excised, measured, imaged and collected for Western blot.

### Immunohistochemistry (IHC) test

A total of 66 patients underwent surgery at the Department of Hepatobiliary Surgery Institute, Southwest Hospital, Third Military Medical University, China, for HCC from 2013 to 2014. Expression profile of FXR and SOCS3 and activation of STAT3 on formalin-fixed paraffin-embedded clinical samples were investigated by IHC. For immunohistochemical staining and scoring, previously described protocols were followed [47]. The immunohistochemical grade was quantified according to the proportion of stained cells into 4 bins as 0–3+ (0: no expression; 1+: weak expression; 2+: moderate expression and 3+: strong expression) (seen in Supplementary Fig. S1). For statistical analysis, as well as to reduce intraobserver variability, the immunohistochemical scores were further grouped into two categories: low (grade 0 or 1+) or high (grade 2+ or 3+). The primary antibody was replaced by rabbit IgG (isotype control) in negative control (NC) sections.

### Statistical analysis

Data were analysed using SPSS 17.0 or GraphPad Prism. When two groups were compared, Student's *t* test was used. When more than two groups wre compared, one-way ANOVA followed by Tukey's Test was carried out. *P* value <0.05 was taken to be statistically significant.

## SUPPLEMENTARY MATERIALS FIGURE


