# Supplementary material for: FXR induces SOCS3 and suppresses hepatocellular carcinoma
Source: Oncotarget. 2015 Sep 21;6(33):34606–16. doi: 10.18632/oncotarget.5314 (PMC4741476; doi:10.18632/oncotarget.5314)
Supplement: Supplementary file 1 [file oncotarget-06-34606-s001.pdf]

SUPPLEMENTARY FIGURE

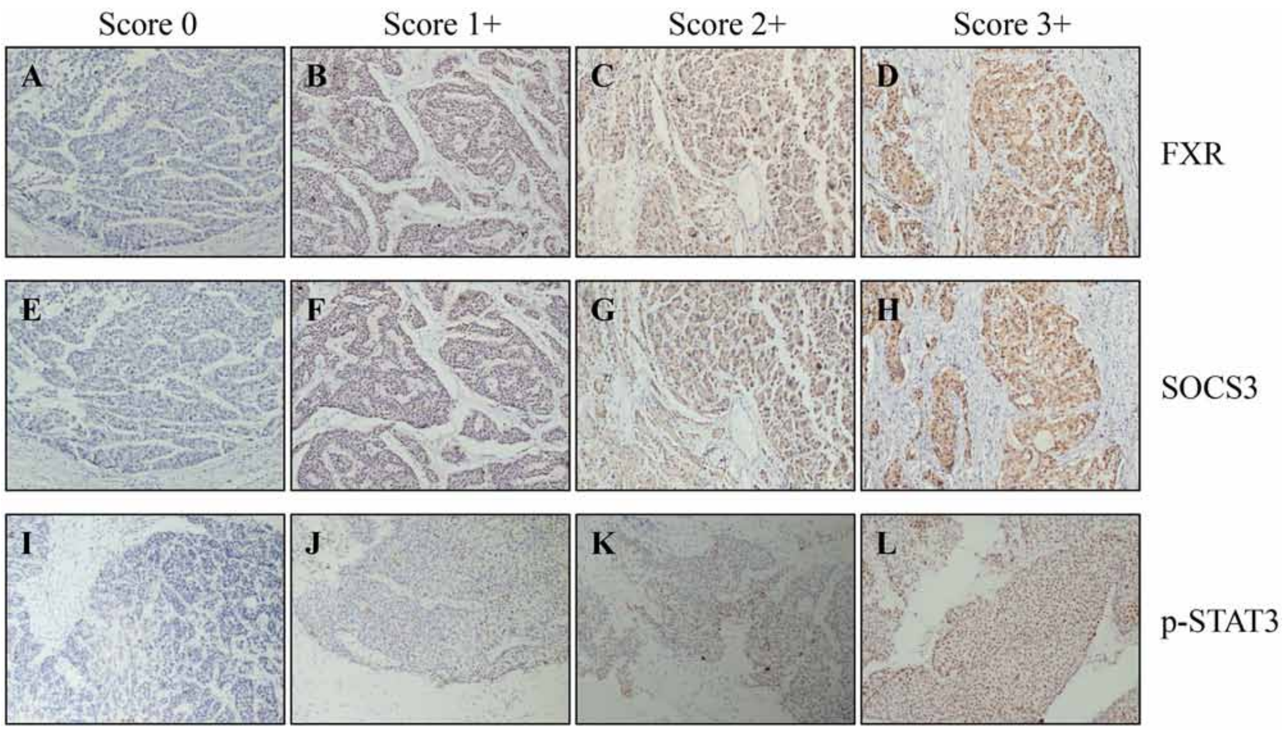

Supplementary Figure S1: Scoring systems for immunohistochemical staining. Scores 0–3+ represent no, weak, moderate and strong expression, respectively. **A–D.** Representative immunohistochemical stainings for FXR in HCC tumoral tissues. **E–H.** Representative immunohistochemical stainings for SOCS3 in HCC tumoral tissues. **I–L.** Representative immunohistochemical stainings for phosphorylated STAT3 (p-STAT3) in HCC tumoral tissues.
